# Usher Syndrome Belongs to the Genetic Diseases Associated with Radiosensitivity: Influence of the ATM Protein Kinase

**DOI:** 10.3390/ijms23031570

**Published:** 2022-01-29

**Authors:** Joëlle Al-Choboq, Mélanie L. Ferlazzo, Laurène Sonzogni, Adeline Granzotto, Laura El-Nachef, Mira Maalouf, Elise Berthel, Nicolas Foray

**Affiliations:** 1 Inserm, U1296 Unit, Radiation: Defense, Health and Environment, Centre Léon-Bérard, 28 rue Laennec, 69008 Lyon, France; joelle.al-choboq@inserm.fr (J.A.-C.); melanie.ferlazzo@inserm.fr (M.L.F.); laurene.sonzogni@inserm.fr (L.S.); adeline.granzotto@inserm.fr (A.G.); laura.el-nachef@inserm.fr (L.E.-N.); elise.berthel@inserm.fr (E.B.); 2 Department of Chemistry and Biochemistry, Faculty of Sciences II, Lebanese University, Fanar 1202, Lebanon; mira.n.maalouf@gmail.com

**Keywords:** Usher syndrome, radiosensitivity, DNA double-strand breaks, ATM, ionizing radiation

## Abstract

Usher syndrome (USH) is a rare autosomal recessive disease characterized by the combination of hearing loss, visual impairment due to retinitis pigmentosa, and in some cases vestibular dysfunctions. Studies published in the 1980s reported that USH is associated with cellular radiosensitivity. However, the molecular basis of this particular phenotype has not yet been documented. The aim of this study was therefore to document the radiosensitivity of USH1—a subset of USH—by examining the radiation-induced nucleo-shuttling of ATM (RIANS), as well as the functionality of the repair and signaling pathways of the DNA double-strand breaks (DSBs) in three skin fibroblasts derived from USH1 patients. The clonogenic cell survival, the micronuclei, the nuclear foci formed by the phosphorylated forms of the X variant of the H2A histone (ɣH2AX), the phosphorylated forms of the ATM protein (pATM), and the meiotic recombination 11 nuclease (MRE11) were used as cellular and molecular endpoints. The interaction between the ATM and USH1 proteins was also examined by proximity ligation assay. The results showed that USH1 fibroblasts were associated with moderate but significant radiosensitivity, high yield of micronuclei, and impaired DSB recognition but normal DSB repair, likely caused by a delayed RIANS, suggesting a possible sequestration of ATM by some USH1 proteins overexpressed in the cytoplasm. To our knowledge, this report is the first radiobiological characterization of cells from USH1 patients at both molecular and cellular scales.

## 1. Introduction

Usher syndrome (USH) is the most frequent cause of combined deafness and blindness in humans. USH is a very rare genetic disorder that combines congenital sensorineural hearing loss of variable severity, retinitis pigmentosa (RP) and, in some cases, vestibular dysfunctions [1,2]. While pigmentary changes in the retinas of some deaf children were first reported by the German researchers Albert Von Gräfe [3] and Richard Liebreich [4,5] before 1860, history retained the name of the Scottish ophthalmologist Charles Howard Usher, who published a princeps paper about the transmission of the disease from 69 cases in 1914 [6].

USH is considered to be an autosomal recessive disease with a prevalence between 3 and 17 in 100,000 individuals [7,8]. Three USH subtypes have been described—USH1, USH2, and USH3—based on the degree of hearing loss, the age of onset of RP, and the presence of vestibular dysfunction [9]. USH1, which accounts for ~35% of USH cases, is the most severe USH subtype, with severe-to-profound congenital hearing loss and prepubertal onset of RP [10]. The different forms of USH1 are caused by mutations of at least six genes [10,11,12], encoding the actin-based motor myosin VIIA protein (USH1B/MYO7A) [13], the harmonin protein (USH1C/Harmonin) [14], two cadherin-related proteins—cadherin-23 (USH1D/CDH23) [15] and protocadherin-15 (USH1F/PCDH15) [16]—and the scaffold protein containing ankyrin repeats and sterile alpha motif (SAM) domain (USH1G/SANS) [17]. The locus previously called USH1A does not exist [18].

In the 1980s, skin fibroblasts derived from USH patients were reported to be radiosensitive in vitro [19,20] (Table 1). Although these findings suggest the occurrence of some complications during or after anticancer radiotherapy, USH patients have not necessarily been subjected to radiotherapy, since USH has not been specifically associated with high cancer proneness. Nevertheless, it is noteworthy that RP can be associated with several fundus tumors, including giant drusen-resembling astrocytoma [21] or rectal adenocarcinoma [22]. Furthermore, some adverse tissue reactions following radiotherapy against laryngeal cancer have been reported in one USH patient [23]. Hence, the response of USH patients to ionizing radiation (IR) may represent both a scientific and medical issue. Further investigations about the radiosensitivity of USH cells are important inasmuch as this syndrome has been classified among the 10 genetic syndromes associated with the highest radiosensitivity in humans [24]. Unfortunately, no mechanistic model has yet been proposed to explain the potential abnormal response to IR associated with USH.

There is increasing evidence that the repair and signaling of the DNA double-strand breaks (DSBs)—the radiation-induced DNA damage the most strongly linked to the radiation-induced (RI) cell death [25]; however, the aforementioned USH proteins are not known to be involved in these processes. Moreover, the USH proteins are mostly localized in the cytoplasm, which contradicts the current paradigm that the radiosensitivity phenotype should be associated with the defect of DSB repair and signaling, whose proteins are generally localized in the nucleus [25]. Some other genetic syndromes have been already reported to be associated with both cellular radiosensitivity and mutations of cytoplasmic proteins; this is notably the case of Huntington’s disease (HD) [26], tuberous sclerosis (TSC) [27], neurofibromatosis type 1 (NF1) [28], and a subset of xeroderma pigmentosum D (XPD) [29]. In these diseases, abnormal response to IR was found to be generated by an overexpression of the cytoplasmic forms of the mutated protein causing the disease and its capacity to bind ATM—a major kinase activated in the early steps of the non-homologous end-joining (NHEJ), which is the predominant DSB signaling and repair pathway in humans.

The link between an abnormal response to IR and the overexpression of some ATM substrates localized in the cytoplasm has been integrated in a mechanistic model based on the radiation-induced ATM nucleo-shuttling (RIANS). Relevant for both high- and low-dose radiobiological phenomena, the RIANS model has been proposed to describe, predict, and quantify the risk linked to any exposure to IR [15,16,17]. In the framework of the RIANS model, the oxidative stress caused by IR induces the monomerization of the ATM dimers in the cytoplasm. The resulting ATM monomers diffuse in the nucleus and phosphorylate the H2AX histone variant (γH2AX) at DSB sites, triggering the formation of nuclear γH2AX foci, as easily quantifiable by immunofluorescence. The formation of nuclear γH2AX foci is considered to be an early recognition step of the RI DSBs repaired by NHEJ [7,15,16,17,18,19,20]. During the DSB repair process, two ATM monomers reassociate on the DSB sites and form the nuclear trans-autophosphorylated ATM (pATM) foci, which are also visible via immunofluorescence. Any delay in the RIANS leads to a radiosensitivity phenotype. By considering the nuclear γH2AX and pATM foci as endpoints, the early (10 min, 1 h) post-irradiation time data provide information about the DSB recognition process, while the late (24 h) post-irradiation time data characterize the DSB repair step [16,21]. The RIANS model provides a statistically robust prediction of post-radiotherapy radiosensitivity [30,31,32,33,34] and a unified model to explain radiosensitivity in a number of genetic syndromes, including HD, TSC, NF1, and XPD [26,27,28,29]. In the framework of the RIANS model and its related biomarkers, this study aims to provide—for the first time to our knowledge—a radiobiological characterization of USH1 fibroblasts.

## 2. Results

### 2.1. Cellular Radiosensitivity of USH1 Fibroblasts

**Figure 1:**
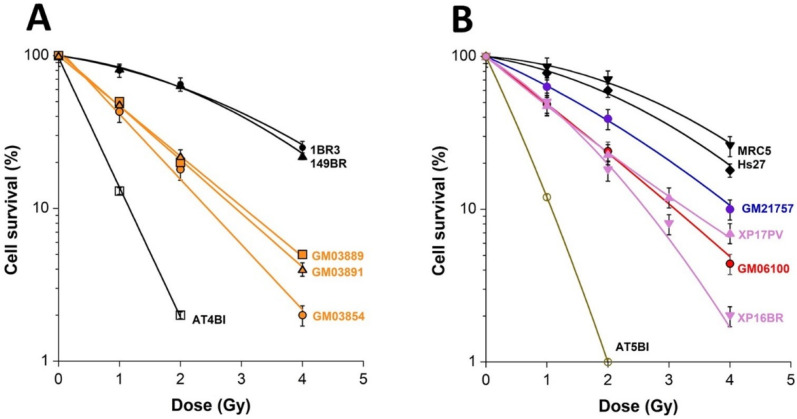
Clonogenic cell survival in USH1 fibroblasts: A clonogenic cell survival assay was applied to the radioresistant controls 1BR3 and 149BR, the hyper-radiosensitive *ATM*-mutated AT4BI, and the three USH1 (GM03854, GM03889, and GM03891) fibroblast cell lines (**A**), and to the radioresistant controls MRC5 and Hs27, the hyper-radiosensitive *ATM*-mutated AT5BI, the HD (GM21757), the TSC (GM06100), and two XPD (XP16BR and XP17PV) fibroblast cell lines (**B**). Each plot represents the mean ± SEM of three replicates. Survival data were fitted to the LQ model.

In order to evaluate the cellular radiosensitivity of USH1, the clonogenic survival assay was applied to three USH1 fibroblast cell lines (GM03854, GM03889, and GM03891), but also to four radioresistant (1BR3, 149BR, MRC5, and Hs27), two hyper-radiosensitive *ATM*-mutated (AT4BI and AT5BI), one HD (GM21757), one TSC (GM06100), and two XPD (XP16BR and XP17PV) fibroblast cell lines (Figure 1).

The cellular radiosensitivity assessed in USH1 fibroblasts appeared to be intermediate—higher than that of radioresistant cells but lower than that of the hyper-radiosensitive fibroblasts. By using the cell survival fraction at 2 Gy (SF2) values as a quantitative parameter of radiosensitivity, the SF2 values of the GM03854, GM03889, and GM03891 cells were found to be 18 ± 3%, 20 ± 1%, and 22 ± 3%, respectively, while the average SF2 values of the radioresistant and hypersensitive controls were 65 ± 3% and 2.2 ± 1.0%, respectively. These findings support a moderate but significant radiosensitivity of the USH1 cells tested—significantly higher (*p* < 0.01) than that of radioresistant, controls but significantly lower (*p* < 0.02) than that of hyper-radiosensitive cells (Figure 1A,B). The LQ parameters of GM03854, GM03889, and GM03891 cells were α = 0.81 ± 0.02 Gy^−1^ and β = 0.029 ± 0.01 Gy^−2^, α = 0.66 ± 0.07 Gy^−1^ and β = 0.052 ± 0.04 Gy^−2^. and α = 0.71 ± 0.01 Gy^−1^ and β = 0.023 ± 0.01 Gy^−2^ (r = 0.99 for the three data fits), respectively. Interestingly, the cellular radiosensitivity of USH1 fibroblasts was found to be in the same range of that of HD, TSC, and XPD cells, in agreement with published reports [26,27,29] (Figure 1B).

### 2.2. Abnormally High Levels of Micronuclei in USH1 Fibroblasts

**Figure 2:**
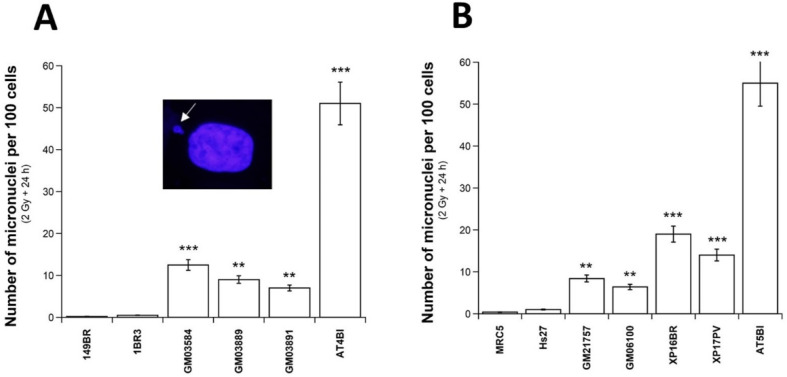
Micronuclei in USH1 fibroblasts: Number of micronuclei per 100 cells assessed 24 h after 2 Gy X-rays in the radioresistant controls 1BR3 and 149BR, the hyper-radiosensitive *ATM*-mutated AT4BI, and the three USH1 (GM03854, GM03889, and GM03891) fibroblast cell lines (**A**), and in the radioresistant controls MRC5 and Hs27, the hyper-radiosensitive *ATM*-mutated AT5BI, the HD (GM21757), the TSC (GM06100), and two XPD (XP16BR and XP17PV) fibroblast cell lines (**B**). Each plot represents the mean ± SEM of three replicates. The insert shows a representative example of a micronucleus observed via DAPI counterstaining. Asterisks represent the statistically significant differences from radioresistant controls, expressed as *p*-values (2 and 3 asterisks correspond to *p* < 0.01 and *p* < 0.001, respectively).

Micronuclei lead to irreversibly damaged chromosomal fragments, causing mitotic death. Micronuclei generally result from the propagation of unrepaired DSBs all along the cell cycle, and may lead to lagging chromosomes or metaphase bridges. Although these latter two specific cytogenetic events have been observed in USH1 cells, the fact that the great majority of cells were irradiated in G0/G1 precluded a robust analysis of the origin of each micronucleus observed. Conversely, whatever their origin, micronuclei have been shown to be quantitatively correlated with cellular radiosensitivity [25], and can be quantified thanks to the 4′,6′diamidino-2-phenyl-indole (DAPI) counterstaining under the same conditions as all of the immunofluorescence data (see Section 4). The number of micronuclei was assessed before or after 2 Gy followed by 24 h in USH1 cells. With regard to spontaneous micronuclei, no significant difference was found between USH1 cells and radioresistant controls (*p* > 0.05), while the number of spontaneous micronuclei per 100 cells was found to be significantly higher in the two hyper-radiosensitive ATM-mutated cells (*p* < 0.001) (data not shown). With regard to micronuclei assessed 24 h post-irradiation, the number of micronuclei per 100 cells was found to be significantly higher (*p* < 0.01) than that of radioresistant controls, but lower (*p* < 0.001) than that observed in the hyper-radiosensitive *ATM*-mutated cells (*p* < 0.04) (Figure 2A), supporting a significant genomic instability in the USH1 cells tested. Again, the data obtained from the HD, TSC, and XPD cells were in the same range as those from the USH1 cells (Figure 2B).

Our previous studies found a quantified correlation between SF2 and micronuclei assessed after 2 Gy, followed by 24 h of repair, in a collection of human fibroblasts showing a wide range of radiosensitivity and representing ~20 genetic diseases [26,27,28,29,35]. The USH1 data were found to be in full agreement with the formula linking micronuclei to SF2 values, supporting the quantitative relevance of our findings (Figure S1).

### 2.3. Abnormal Number of γH2AX Foci after Irradiation in USH1 Fibroblasts

**Figure 3:**
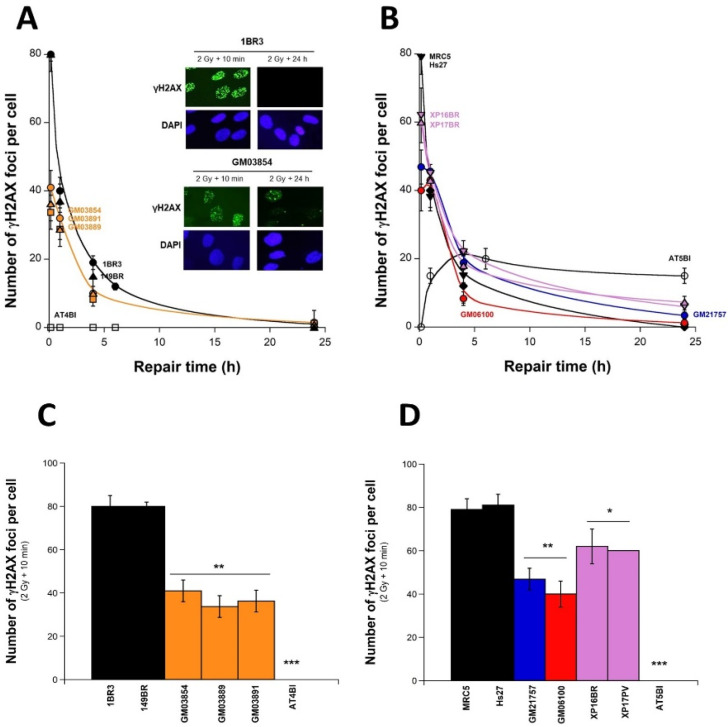
Kinetics of the appearance/disappearance of γH2AX foci in USH1 fibroblasts: The number of γH2AX foci was plotted against post-irradiation time. Data were obtained from the radioresistant controls 1BR3 and 149BR, the hyper-radiosensitive *ATM*-mutated AT4BI, and the three USH1 (GM03854, GM03889, and GM03891) fibroblast cell lines (**A**), and from the radioresistant controls MRC5 and Hs27, the hyper-radiosensitive *ATM*-mutated AT5BI, the HD (GM21757), the TSC (GM06100), and two XPD (XP16BR and XP17PV) fibroblast cell lines (**B**). Panels (**C**,**D**) represent the 10 min post-irradiation data only. Each plot represents the mean ± SEM of three replicates. Data were fitted to Bodgi’s formula [36]. The insert shows a representative illustration of DAPI-stained nuclei and γH2AX foci observed at 10 min and 24 h post-irradiation (2 Gy X-rays) in the indicated cell lines. Asterisks represent statistically significant differences from radioresistant controls, expressed as *p*-values (1, 2, and 3 asterisks correspond to *p* < 0.05, *p* < 0.01, and *p* < 0.001, respectively).

Since micronuclei result from unrepaired DSBs, the recognition and repair of radiation-induced DSBs were investigated in USH1 fibroblasts. The kinetics of appearance/disappearance of nuclear γH2AX foci provide information about DSB recognition (early foci) and repair (late foci) [34]. Under our conditions, even if some spontaneous γH2AX foci were observed in the three USH1 fibroblasts (GM03889: 0.3 ± 0.1, GM03854: 0.4 ± 0.07, and GM03891: 0.3 ± 0.09 γH2AX foci per cell), their number was not significantly different from in the radioresistant controls (0.2 ± 0.3 on average; *p* > 0.1; Figure 3). In the radioresistant controls, the number of γH2AX foci measured 10 min after 2 Gy was 79 ± 4 per cell, in very good agreement with the value of 37 ± 4 per Gy per cell published previously [35]. The number of induced γH2AX foci measured 10 min post-irradiation in the three USH1 fibroblasts was systematically lower than in the radioresistant controls (GM03889: 33.6 ± 6.9, GM03854: 41 ± 5.7, and GM03891: 36.3 ± 3.8 γH2AX foci per cell; *p* < 0.01) (Figure 3A,B). 

In order to better illustrate these differences, the histograms shown in Figure 3C,D reproduce the 10 min data only. No difference was found between the size of nuclei in USH1 and control cells. Our findings therefore suggest an impairment in the DSB recognition. At 24 h post-irradiation, the USH1 fibroblasts showed systematically more γH2AX foci 24 h post-irradiation (GM03889: 1.2 ± 0.2, GM03854: 1.5 ± 0.4, and GM03891: 0.9 ± 0.1 γH2AX foci per cell) than the radioresistant controls (0 ± 0.5 γH2AX foci per cell); however, these differences were at the limit of significance (*p* = 0.05) (Figure 3A,B). Altogether, these data suggest a lack of complete DSB recognition without any significant DSB repair defect. Since cellular radiosensitivity associated with no significant DSB repair defect has already been reported in other genetic syndromes—notably in the HD, TSC, NF1, and XPD fibroblasts [26,27,28,29]—we examined the γH2AX foci kinetics in cells derived from these syndromes. The HD, TSC, and XPD cells showed γH2AX foci kinetics similar to those obtained with the USH1 cells tested, as with survival and micronuclei data (Figure 3B). 

In order to once again consolidate the whole quantitative coherence of our USH1 data, the γH2AX foci and the corresponding SF2 data shown in Figure 1 were plotted on the same graph (Figure S2). The SF2 and γH2AX data obtained from the three USH1 fibroblasts (GM03854, GM03889, and GM03891) were found to be in good agreement with the quantitative correlation obtained from a collection of human fibroblasts showing a wide range of radiosensitivity and representing ~20 genetic syndromes [26,27,28,29,35] (Figure S2).

### 2.4. Abnormal Number of pATM Foci after Irradiation in USH1 Fibroblasts

**Figure 4:**
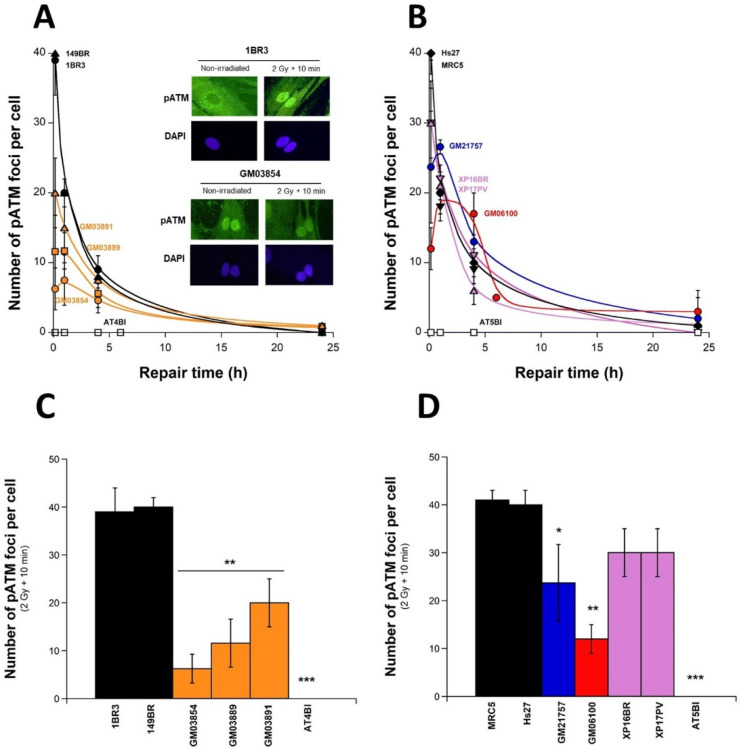
Kinetics of the appearance/disappearance of pATM foci in USH1 fibroblasts: The number of pATM foci was plotted against post-irradiation time. Data were obtained from the radioresistant controls 1BR3 and 149BR, the hyper-radiosensitive *ATM*-mutated AT4BI, and the three USH1 (GM03854, GM03889, and GM03891) fibroblast cell lines (**A**), and from the radioresistant controls MRC5 and Hs27, the hyper-radiosensitive *ATM*-mutated AT5BI, the HD (GM21757), the TSC (GM06100), and two XPD (XP16BR and XP17PV) fibroblast cell lines (**B**). Panels (**C**,**D**) represent the 10 min post-irradiation data only. Each plot represents the mean ± SEM of three replicates. Data were fitted to Bodgi’s formula [36]. The insert shows a representative illustration of DAPI-stained nuclei and pATM foci observed at 10 min post-irradiation (2 Gy X-rays) in the indicated cell lines. Asterisks represent the statistically significant differences from radioresistant controls, expressed as *p*-values (1, 2, and 3 asterisks correspond to *p* < 0.05, *p* < 0.01, and *p* < 0.001, respectively).

As described above, the number of induced γH2AX foci measured 10 min after irradiation in USH1 fibroblasts was found to be systematically lower than in the radioresistant controls. As previously described, these data do not suggest that fewer DSBs were physically induced in USH1 cells but, rather, that fewer DSBs were recognized by the NHEJ pathway [34]. Based on our historical data, an exposure to 2 Gy X-rays generally results in the formation of ~40 pATM foci per cell at 10 min post-irradiation in radioresistant controls. This number progressively decreased with repair time, and was not found to be different from zero at 24 h post-irradiation [29]. In this study, data obtained with the radioresistant control cell line reached similar conclusions (44 ± 8 and 1 ± 2 pATM foci per cell at 10 min and 24 h post-irradiation, respectively) (Figure 4A,B). In order to better illustrate these differences, the histograms shown in Figure 4C,D reproduce the 10 min data only.

In USH1 fibroblasts, the number of pATM foci per cell observed 10 min post-irradiation was found to be significantly lower than in control fibroblasts (GM03889: 11.7 ± 7.4, GM03891: 20 ± 0, and GM03854: 6.3 ± 3.4 and 1BR3: 44 ± 8; *p* < 0.001). A similar conclusion was reached with pATM foci data assessed at 1 h post-irradiation, in agreement with γH2AX data, and supporting the notion that the USH1 fibroblasts elicited abnormal ATM nuclear kinase activity in response to radiation (Figure 4A). The HD, TSC, and XPD cells also showed lower numbers of early pATM foci than in radioresistant controls (Figure 4B).

In order to once again consolidate the whole quantitative coherence of our USH1 data, the pATM foci obtained 10 min post-irradiation and the corresponding SF2 data shown in Figure 1 were plotted on the same graph (Figure S3). The SF2 and the early pATM data obtained from the three USH1 fibroblasts (GM03854, GM03889, and GM03891) were found to be in agreement with the quantitative correlation obtained from a collection of human fibroblasts showing a wide range of radiosensitivity, and representing ~20 genetic diseases [26,27,28,29,35] (Figure S3).

In USH1 fibroblasts, the number of pATM foci per cell observed 10 min post-irradiation was found to be significantly lower than in control fibroblasts (GM03889: 11.7 ± 7.4, GM03891: 20 ± 0; GM03854: 6.3 ± 3.4, and 1BR3: 44 ± 8; *p* < 0.001). A similar conclusion was reached with pATM foci data assessed at 1 h post-irradiation, in agreement with γH2AX data, and supporting the notion that the USH1 fibroblasts elicited abnormal ATM nuclear kinase activity in response to radiation (Figure 4A). The HD, TSC, and XPD cells also showed lower numbers of early pATM foci than in radioresistant controls (Figure 4B). In order to consolidate the whole quantitative coherence of our USH1 data, the pATM foci obtained 10 min post-irradiation and the corresponding SF2 data shown in Figure 1 were plotted on the same graph (Figure S3). The SF2 and the early pATM data obtained from the three USH1 fibroblasts (GM03854, GM03889, and GM03891) were found to be in agreement with the quantitative correlation obtained from a collection of human fibroblasts showing a wide range of radiosensitivity and representing ~20 genetic diseases [26,27,28,29,35] (Figure S3).

### 2.5. Abnormal Number of MRE11 Foci after Irradiation in USH1 Fibroblasts

**Figure 5:**
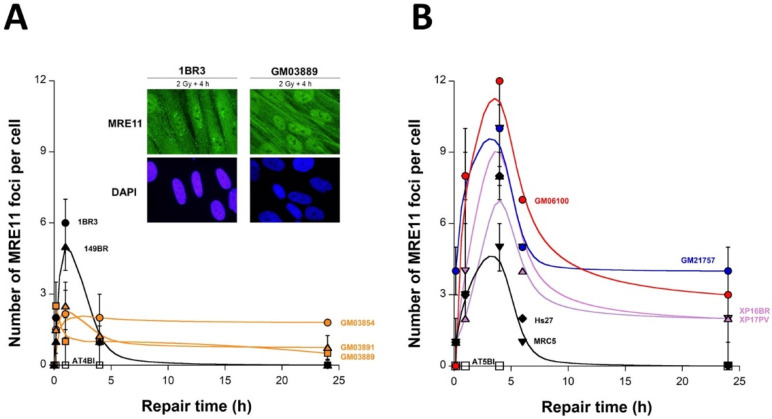
Kinetics of the appearance/disappearance of MRE11 foci in USH1 fibroblasts: The number of MRE11 foci was plotted against post-irradiation time. Data were obtained from the radioresistant controls 1BR3 and 149BR, the hyper-radiosensitive *ATM*-mutated AT4BI, and the three USH1 (GM03854, GM03889, and GM03891) fibroblast cell lines (**A**), and from the radioresistant controls MRC5 and Hs27, the hyper-radiosensitive *ATM*-mutated AT5BI, the HD (GM21757), the TSC (GM06100), and two XPD (XP16BR and XP17PV) fibroblast cell lines (**B**). Each plot represents the mean ± SEM of three replicates. Data were fitted to Bodgi’s formula [36]. The insert shows a representative illustration of DAPI-stained nuclei and MRE11 foci observed at 4 h post-irradiation in the indicated cell lines.

The MRE11 protein is a component of the RAD50-MRE11-NBS1 complex that forms nuclear foci after genotoxic stress. The formation of MRE11 foci after irradiation appeared to be impaired in the ataxia telangiectasia cells tested with the same defined pattern of MRE11 foci [35,37]. To consolidate the observation of an impairment of the ATM activity in USH1 cells, we investigated the occurrence of MRE11 foci in irradiated USH1 fibroblasts. The MRE11 foci in the radioresistant controls appeared from 2 to 8 h post-irradiation, and reached their maximal yield at 4 h (7 ± 2 MRE11 foci per cell) (Figure 5A,B). The shape of the MRE11 foci kinetics of the three USH1 fibroblasts appeared to be clearly different from that of the radioresistant controls. The maximal number of MRE11 foci was reached at 10 min (for GM03889), 1 h (for GM03891), or 4 h post-irradiation, but never reached the maximal value assessed in the radioresistant controls. At 4 h post-irradiation, the values were GM03889: 1 ± 0.8, GM03854: 2.2 ± 1.2, and GM03891: 1.3 ± 1.3 vs. 1BR3: 7 ± 2, *p* > 0.05 (Figure 5A). At 24 h post-irradiation, the number of MRE11 foci was found to be different from zero for the three USH1 cell lines tested, and was higher than for controls, even if this trend was statistically significant only for the GM03854 cells (*p* < 0.01). Altogether, these findings indicate that the USH1 fibroblasts tested elicited an abnormal MRE11 foci formation after radiation. The MRE11 foci kinetics obtained from the HD, TSC, and XPD cells are also shown in Figure 5B. The data obtained from the HD, TSC, and XPD fibroblasts tested showed significantly higher numbers of MRE11 at 1 h and 4 h post-irradiation (*p* < 0.05), and a higher number of MRE11 foci at 24 h post-irradiation (*p* < 0.1), compared to USH1 data, suggesting a different involvement of MRE11 nuclease in the response of USH1 cells to IR than in the other syndromes tested (Figure 4B). 

It is noteworthy that no correlation was obtained between the SF2 data and the MRE11 data from any of the cell lines tested, whatever the post-irradiation time (data not shown), confirming again that the MRE11 foci are not reliable endpoints for predicting radiosensitivity, as reported previously [37].

### 2.6. Subcellular Localization and Expression of the USH1 Proteins in USH1 Fibroblasts

**Figure 6:**
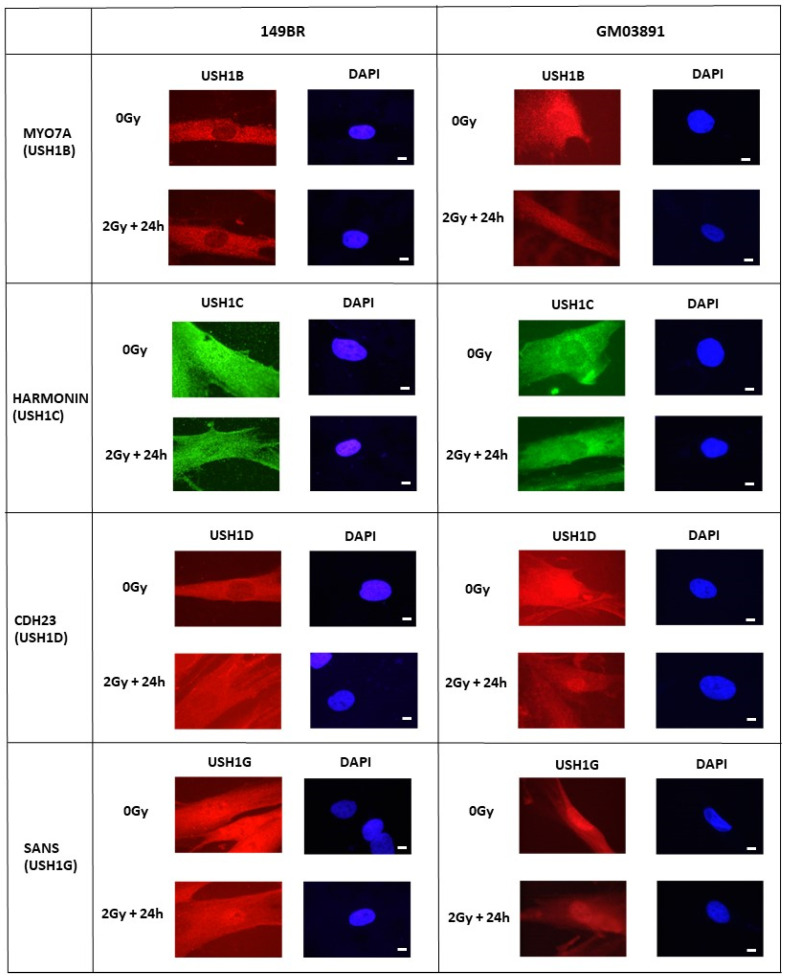
Subcellular localization of USH1 proteins in USH1 and control fibroblasts: Anti-USH1B/MYO7A, anti-USH1D/CDH23, anti-USH1C/Harmonin, and anti-USH1G/SANS immunofluorescence was applied to the radioresistant 149BR controls and the USH1 GM03891 fibroblasts, either before or after irradiation (2 Gy + 24 h), as indicated. The white bar represents 5 µm.

Cellular radiosensitivity has been generally linked to impairment in DSB repair and signaling, associated with dysfunction of proteins that are generally nuclear [25]. To further investigate the role of the USH1 proteins in the molecular response to IR, their subcellular localization was analyzed by applying immunofluorescence as a first step. Because the mutations of USH1B, USH1C, USH1D, and USH1G represent the great majority of USH1 cases (see Section 1), we focused on these four proteins. The radioresistant control fibroblasts showed USH1 protein signals as being essentially localized in the cytoplasm, with the exception of some nuclear forms encountered in USH1C/Harmonin and USH1G/SANS (Figure 6). X-ray irradiation did not change this conclusion, regardless of the repair time investigated. In all of the USH1 fibroblasts tested, the USH1B/MYO7A, USH1C/Harmonin, USH1D/CDH23, and USH1G/SANS proteins appeared to be essentially localized in the cytoplasm before irradiation. Some nuclear forms of USH1C/Harmonin and USH1G/SANS were also observed; again, X-rays did not change the subcellular localization of these proteins in the three USH1 fibroblasts tested (Figure 6). Altogether, these data suggest that all of the USH1 proteins tested are expressed in the cytoplasm of USH1 fibroblasts.

**Figure 7:**
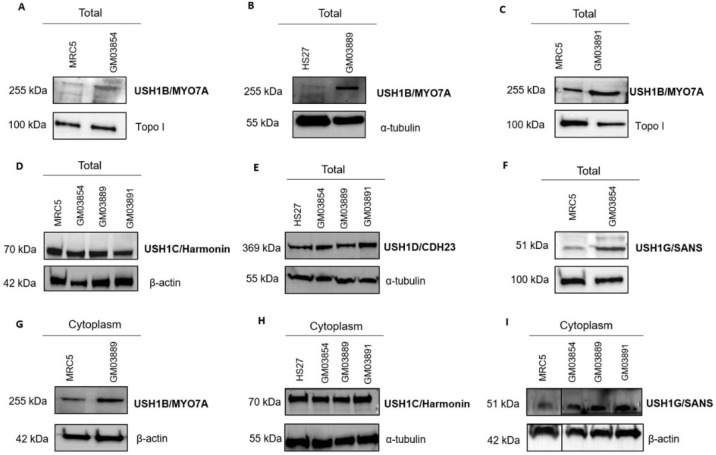
Representative immunoblots of USH1 protein expression in USH1 fibroblasts: A total of 30 µg of protein was subjected to SDS/PAGE. Representative immunoblots showing the expression of different USH1 proteins in the indicated radioresistant control and USH1 fibroblasts. Expression of USH1B (**A**–**C**), USH1C (**D**), USH1D (**E**), and USH1G (**F**) in total protein extracts. Expression of USH1B (**G**), USH1C (**H**), and USH1G/SANS (**I**) in cytoplasmic protein extracts. It is noteworthy that the blot was cut to provide panel I.

Since simple immunofluorescence analysis does not enable quantitative evaluation of the amount of protein, we examined the expression of the USH1B, USH1C, USH1D, and USH1G proteins by using immunoblots. All of the tested USH1 proteins migrated at the expected size in the radioresistant controls and in the USH1 fibroblasts, eliminating the possibility of truncated mutations (Figure 7). The USH1B/MYO7A and USH1G/SANS proteins appeared to be significantly more expressed in USH1 fibroblasts than in radioresistant controls. Immunoblots with cytoplasmic extracts reached similar conclusions, suggesting an overexpression of the USH1B/MYO7A and USH1G/SANS proteins in the cytoplasm of the three USH1 fibroblasts tested (Figure 7 and data not shown). It should be noted that both USH1 GM03889 and GM03891 cells were found to have mutations in the *USH1B* gene, and no *USH1* mutation has yet been identified in USH1 GM03854 cells, but not all of the promoter sequence of USH1B has been investigated (see Section 4).

### 2.7. Some USH1 Proteins Interact with ATM More Abundantly in USH1 Fibroblasts

**Figure 8:**
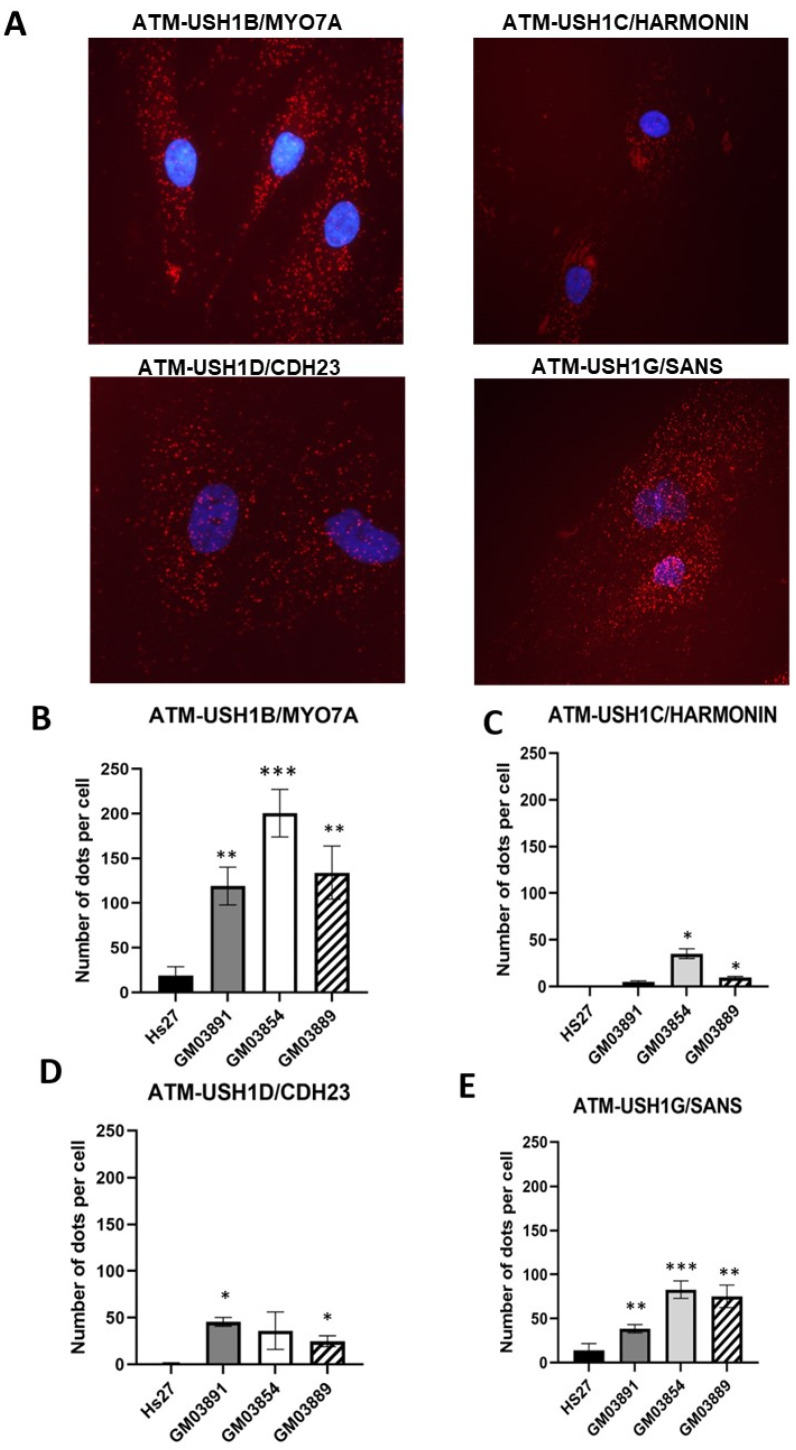
Interaction between ATM and USH1 proteins: A proximity ligation assay (PLA) was applied to the indicated cell lines. Representative PLA images obtained from GM03891 cells (**A**). Some nuclear PLA signals may be observed, but they do not represent the majority of ATM-USH1 protein complexes. The average numbers of red dots representing the cytoplasmic ATM-USH1 protein complexes were scored per 100 cells (**B**–**E**). Each datum represents the mean ± SEM of at least two independent replicates. The nuclei were counterstained with DAPI (blue). Asterisks represent the statistically significant differences from radioresistant controls, expressed as *p*-values (1, 2 and 3 asterisks correspond to *p* < 0.05, *p* < 0.01, and *p* < 0.001, respectively).

Some USH1 proteins, such as USH1B/MYO7A, hold putative SQ and/or TQ domains of phosphorylation by ATM [38], suggesting that these proteins may interact with ATM and be phosphorylated. Since the RIANS is delayed in the USH1 fibroblasts, and since these USH1 proteins appeared to be overexpressed in the cytoplasm of USH1 fibroblasts, we hypothesized that some USH1-ATM protein complexes may be formed after irradiation, consistent with the delayed RIANS. Similar hypotheses have notably been presented in HD, TSC, NF1, and XPD syndromes [26,27,28,29]. We therefore, as a first step, examined the existence of these USH1-ATM complexes in USH1 fibroblasts and their subcellular localization. A proximity ligation assay (PLA) was therefore applied on the three USH1 cell lines tested. The images obtained revealed significant red dots in the cytoplasm of the USH1 cell lines, supporting the existence of cytoplasmic USH1B-ATM, USH1C-ATM, USH1D-ATM, and USH1G-ATM complexes whose abundance changed depending on the USH1 cell line tested. Although some USH1-ATM complexes may be found in the nucleus, depending on the nature of the USH1 protein, the number of the USH1-ATM complexes clearly appeared to be much higher in USH1 cells than in control cells—especially in the cytoplasm (Figure 8). Altogether, our findings suggest that ATM binds to some USH1 proteins in the cytoplasm, but to an extent that depends on USH1 individuals. 

### 2.8. Treatment with Statins and Bisphosphonates Protects USH1 Fibroblasts from Radiation

**Figure 9:**
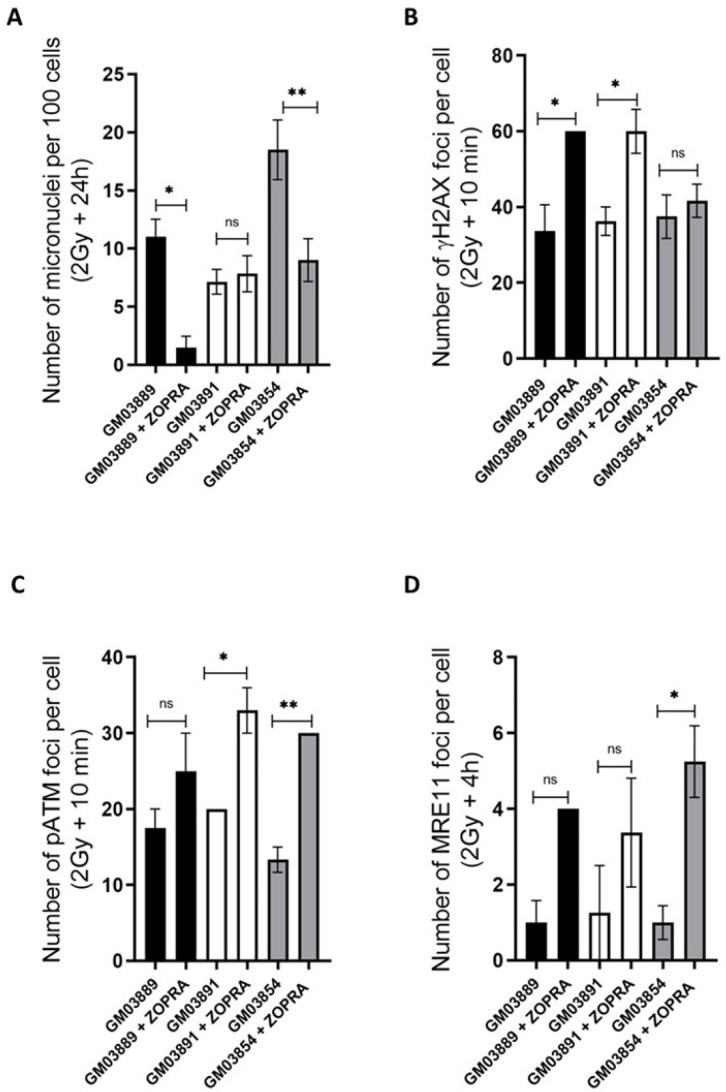
Effect of ZOPRA treatment in USH1 fibroblasts in response to radiation: Numbers of micronuclei per 100 cells assessed after 2 Gy followed by 24 h post-irradiation (**A**), numbers of γH2AX foci per cell assessed after 2 Gy followed by 10 min post-irradiation (**B**), numbers of pATM foci per cell assessed after 2 Gy followed by 10 min post-irradiation (**C**), and numbers of MRE11 foci per cell assessed after 2 Gy followed by 4 h post-irradiation (**D**) in the GM03889, GM03891, and GM03854 USH1 fibroblasts, with or without ZOPRA treatment. Data points represent the mean ± SEM of duplicate experiments. Asterisks represent the statistically significant differences from radioresistant controls, expressed as *p*-values (1, and 2 asterisks correspond to *p* < 0.05, and *p* < 0.01, respectively, ns corresponds to *p* > 0.1).

In previous studies, the combination of pravastatin and zoledronate, known as the ZOPRA treatment, was shown to accelerate the RIANS and to decrease radiosensitivity in a number of genetic diseases, including HD, TSC, NF1, and XPD [26,27,28,29]. The choice of these two drugs was motivated by published findings suggesting that the combination of statins and aminobisphosphonates efficiently inhibits both farnesylation and geranylgeranylation of progerin and prelamin A, and markedly improves nuclear abnormalities associated with DNA damage repair and signaling impairments [39]. The ZOPRA treatment did not significantly affect the yields of micronuclei or the kinetics of γH2AX, pATM, and MRE11 foci in radioresistant control cells (data not shown). With regard to micronuclei, the ZOPRA treatment significantly reduced the yield of micronuclei in USH1 fibroblasts, with the exception of the GM03891 cells (Figure 9A). With regard to the γH2AX data, the number of γH2AX foci assessed 10 min post-irradiation was found to be significantly increased in the GM03889 and GM03891 cells, but not in the GM03954 cells (Figure 9B). With regard to the pATM data, the amount of pATM assessed 10 min post-irradiation increased in all of the USH1 cells tested, but this trend was not significant in GM03889 cells (Figure 9C). Similarly, with regard to the MRE11 data, the amount of MRE11 assessed at 4 h post-irradiation increased in all of the USH1 cells tested, but this trend was not significant in GM03891 cells (Figure 9D). Altogether, these data suggest that the ZOPRA treatment may play a role in protecting USH1 fibroblasts against IR by accelerating the RIANS and stimulating the ATM kinase activity in the nucleus. However, the benefit of the treatment may strongly depend on USH1 individuals and molecular endpoints.

## 3. Discussion

### 3.1. The USH1 Fibroblasts Show Significant Molecular and Cellular Radiosensitivity

The USH1 fibroblasts tested here showed significant radiosensitivity associated with a low SF2 value, a high yield of residual micronuclei, and a significant delay in the RIANS, with abnormal γH2AX, pATM, and MRE11 foci kinetics after irradiation. The ZOPRA data suggest that an acceleration of RIANS may decrease some aspects of the radiosensitivity of USH1, and there is evidence of the existence of abundant USH1-ATM complexes in the cytoplasm of USH1 cells. Hence, the radiosensitivity of USH cells reported in the 1980s [19,20] is confirmed and consolidated by the present study. It must be stressed that the level of this radiosensitivity is among the 10 highest described in humans [24]. However, our findings should be considered at the scale of the number of cases investigated—indeed, even if the different techniques and endpoints independently converge to the same conclusions, we are aware that this report is based on the radiobiological characterization of only three USH1 fibroblast cell lines.

USH1 is the most severe form of USH. It is not possible to clinically discriminate the forms of USH1 caused by the mutations of the different causative *USH1* genes. However, the *USH1B*/*MYO7A* and *USH1D*/*CDH23* mutations represent 53–73% and 7–20% of USH1 cases, respectively [40]. Interestingly, *USH1D*/*CDH23* mutations generally result in truncated proteins (nonsense, frameshift, or splice mutations), while the great majority of *USH1B*/*MYO7A* mutations are missense mutations. Furthermore, the *USH1C* gene is rarely involved in USH1 cases, and no mutation has been identified in the *USH1G/SANS* gene [40]. As evoked in the Materials and Methods, DNA sequencing confirmed the USH1 status for two (GM03889 and GM03891 cells) of the three USH1 cell lines tested here. Further investigations are needed in order to pursue the DNA sequencing analysis of the GM03854 cells, and to document the genotype–phenotype relationship.

### 3.2. USH1 Proteins Interact with ATM in the Cytoplasm and May Influence the RIANS

**Figure 10:**
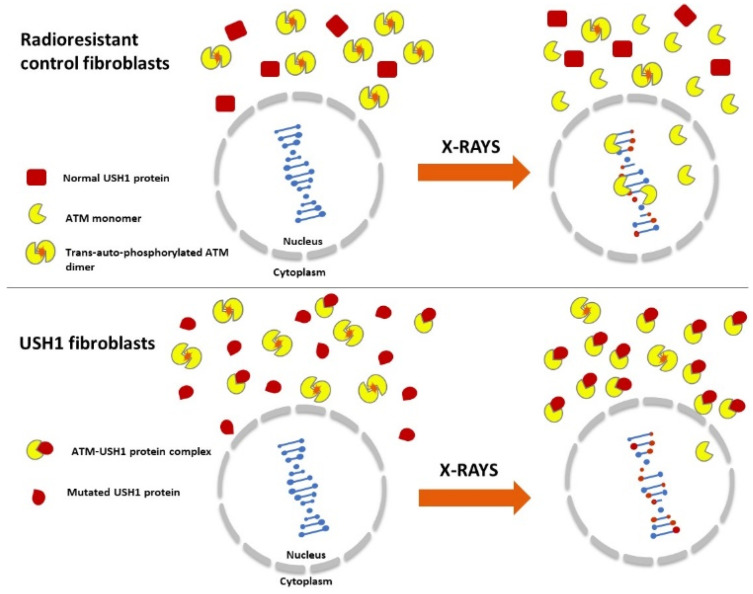
The RIANS model in the USH1 cells: IR induce the monomerization of ATM dimers. The resulting ATM monomers diffuse into the nucleus and participate in DSB recognition via H2AX phosphorylation. In the USH1 cells, USH1 proteins are found to be overexpressed and, as ATM phosphorylation substrates, interact with ATM monomers. The formation of the ATM-USH1 complexes prevents the diffusion of a number of ATM monomers in the nucleus, and delays the ATM nucleo-shuttling, leading to a lack of DSB recognition and to the radiosensitivity phenotype of USH1 cells.

The cellular radiosensitivity linked to USH was first observed in the 1980s [19,20]. To our knowledge, the present study is one of the first examples of investigations of the molecular and cellular responses of USH1 cells to IR. Our approach was based on the RIANS model that has been successfully validated with a number of other genetic diseases [26,27,29,34,41,42,43]. The RIANS model is based on three steps: (1) immediately after irradiation, cytoplasmic ATM dimers dissociate in ATM monomers in a linearly dose-dependent manner; (2) the resulting ATM monomers diffuse in the nucleus; and (3) the ATM monomers phosphorylate the H2AX molecules around the DSB sites, triggering a cascade of ATM-dependent phosphorylation of ATM substrates, from the recognition of DSBs to their complete repair via the NHEJ pathway [30,44]. When these RI steps are rapid (i.e., less than 10 min after 2 Gy X-rays), they lead to a phenotype of radioresistance (called group I). Any delay in the RIANS—especially one caused by the formation of a protein complex with ATM monomers—leads to the impairment of DSB recognition and repair and, therefore, to a phenotype of significant but moderate radiosensitivity (called group II) [44]. In some cases, the DSB recognition (e.g., when *ATM* is mutated) or the DSB repair step (e.g., when *LIG4* is mutated) can be totally prevented, leading to a hyper-radiosensitivity phenotype (called group III) corresponding to an SF2 lower than 20%. The group III syndromes are generally caused by the suppression of a biological function caused by homozygous mutations, while group II syndromes are caused by heterozygous mutations [44]. Interestingly, the USH syndrome is a recessive genetic disorder, which may suggest that USH1 syndrome can be classified as a group III disease, but the SF2 values assessed in the present study in the USH1 cells were found to be between 18 and 22%, i.e., corresponding to the cellular radiosensitivity of the genetic diseases belonging to group II of radiosensitivity, such as HD, TSC, NF1, and XPD [26,27,28,29]. The tested USH1 fibroblasts also showed a delayed RIANS, likely caused by the existence of cytoplasmic USH1-ATM complexes that may facilitate the sequestration of ATM proteins. A delayed RIANS is a specific feature of group II syndromes (Figure 10).

Interestingly, the USH1 proteins are not associated with specific functions in DSB repair and signaling pathways. Moreover, the yields of residual γH2AX foci reflecting unrepaired DSBs found in the tested USH1 fibroblasts were very low, consistent with the absence of any DSB repair defect. However, there are some emerging links between kinetochore and microtubule proteins and USH proteins, but USH2 rather than USH1 proteins may be required for the control of chromatin integrity and the formation of normal metaphases [45]. Conversely, the DSB recognition, reflected by the yield of early γH2AX and pATM foci, was found to be impaired, while the great majority of the cells irradiated in these experiments were quiescent. Our findings therefore suggest that USH1 is also required for the very early response to IR, but this step does not concern the formation of mitoses; the potential role of USH1 in mitotic cells, as “protein”, cannot be incriminated in the DSB recognition process. In parallel, the present findings and the RIANS model suggest that some mutated USH1 proteins are overexpressed “substrates” of ATM (whose requirement in the DSB recognition process is well documented), and contribute to its sequestration in the cytoplasm (Figure 10). There are numerous analogies with other genetic syndromes such as HD, TSC, NF1, and XPD, characterized by moderate but significant radiosensitivity, delayed RIANS, and significant impairment of DSB recognition but normal DSB repair [26,27,28,29]. Like the aforementioned syndromes, USH1 may be associated with mutated protein substrates of ATM that are overexpressed in the cytoplasm [26,27,28,29]. However, further investigations are needed in order to better understand the causes of the overexpression of the USH1 proteins found in cells from USH1 patients. 

### 3.3. USH1 Syndrome, Cancer Proneness, and Aging?

The radiosensitivity of USH1 cells is among the highest reported in humans. However, radiosensitivity does not necessarily mean cancer proneness. For example, progeroid syndromes are in the top three of human radiosensitivity syndromes, but progeria is associated not with cancer proneness but with aging [37]. Considering the potential role of USH1 proteins in genome maintenance and in cellular scaffold integrity, it is important to discuss these two different clinical features.

In our results, a drastic increase in the number of MRE11 foci early after irradiation was generally associated with high cancer proneness. This is notably the case of fibroblasts from neurofibromatosis type I patients [28]. Conversely, when the number of MRE11 foci is lower than controls in the first hour post-irradiation, and progressively increases with post-irradiation time to reach its maximal value at 24 h, it is associated with aging and neurodegeneration [34]. Interestingly, the MRE11 response of the three USH1 cell lines tested here was clearly different from that of the controls, but showed a combination of the two trends described above: two USH1 cell lines showed their highest number of MRE11 foci early after irradiation, but without exceeding those of controls; conversely, the three USH1 cell lines showed more delayed MRE11 foci than controls. In our results, these MRE11 data suggest both the presence of some early misrepaired DNA strand breaks that might reflect a cancer-prone hyper-recombination process, and the accumulation of some late DNA strand breaks that are generally associated with an aging phenotype [37]. Interestingly, by focusing on the particular case of the USH1B/MYO7A protein, it appears that its overexpression promotes cellular proliferation and the lack of adhesion between cells. This is notably the case for some melanoma models, in which the overexpression of the USH1B/MYO7A protein facilitates their progression, which may be a link to cancer proneness [46]. In parallel, mutations in the *USH1B/MYO7A* genes result in the progressive deterioration of hair cells, leading to hearing loss, which may suggest an aging-like process [12]. These two potential consequences of the *USH1* mutations may strongly depend on the nature of the mutation and the mutated protein, but overall on the tissue considered. Such duality may be also compared with some group III diseases, such as ataxia telangiectasia, which combine a high risk of lymphoma and an early degeneration of the Purkinje cells or some subset of XPD showing both skin tumors and neurological defects [47]. Again, further investigations are needed in order to better understand the function of USH1 proteins in the radiosensitivity, radiosusceptibility, and radiodegeneration processes, as well as the phenotype–genotype relationship.

## 4. Materials and Methods

### 4.1. Cell Lines

All of the experiments were performed with untransformed fibroblast cells in the plateau phase of growth, under standard culture conditions described elsewhere [30,48]. All of the fibroblast cell lines used in this study were purchased from commercial repositories. Four radioresistant (1BR3, 149BR, MRC5, and Hs27) controls originating from apparently healthy patients, two hyper-radiosensitive ataxia telangiectasia (*ATM-*mutated; AT4BI and AT5BI), one Huntington’s disease (HD) (GM21757), one tuberous sclerosis syndrome (TSC) (GM06100), two xeroderma pigmentosum D (XPD) (XP16BR and XP17PV), and three USH1 (GM03889, GM03891, and GM03854) fibroblast cell lines were used in this study. The radiobiological features of the non-USH1 cells have been detailed and published elsewhere [26,27,28,29,30,48]. The three USH1 (GM03889, GM03891, and GM03854) fibroblast cell lines were purchased from Coriell Cell Repositories (Camden, NJ, USA). All of these cell lines were derived from male USH1 patients. The age at sampling of the corresponding USH donors was 22, 16, and 47, respectively. With regard to their clinical features provided by the Coriell Institute, both the GM03889 and GM03891 donors suffered from tapetoretinal degeneration, while the GM03854 donor was mildly affected with a slow progression of RP. 

### 4.2. DNA Extraction and MYO7A Sequencing

DNA was extracted using the QIAamp DNA Micro Kit (#56304, QIAGEN, Hilden, Germany). The coding exons and flanking intronic sequences of MYO7A were amplified and sequenced using forward and reverse primers designed using Primer3 (v.0.4.0). Primer sequences and conditions are available upon request. The following primer sequences were used for the amplification of exons 14 (MYO7A_EX14F and MYO7A_EX14R) and 40, (MYO7A_EX40F and MYO7A_EX40R), respectively: GTAGTTCCAATTCATCCACTTAAC, ATGCACAGCACTGTGAAGTACTTAG, AGGTCCTGTGACTCCCGATG, and AGGGGCTCATCCCACAAG, respectively. Sequences were run on an ABI3730 DNA analyzer, and assembled using ABI Prism SeqScape 3.0 from the GenBank reference sequence. Two *USH1B/MYO7A* mutations were detected in GM03889 and GM03891 cells: the 1555-8C > G splice site variant on intron 13 (p.([Gly519Serfs*27,Gly519Alafs*58]). rs1057517774), which has been previously reported in association with USH1 [49], and the 5515_5522dup variant on exon 40 (p.(Gly1842Cysfs*40). rs762117246), which has only been reported in the deafness variation database. Concerning the GM03854 cells, no mutation was detected in the *USH1B/MYO7A*, *USH1C/Harmonin*, or *USH1D/CDH23* gene sequences. 

### 4.3. X-ray Irradiation

Irradiations was performed with a 6 MeV X-ray medical irradiator (SL 15 Philips) (dose rate: 6 Gy.min^−1^) at the Centre Léon-Bérard (Lyon, France) [30,48]. In all of the experiments, a dose of 2 Gy was chosen because it simulates a current dose per session in standard radiotherapy.

### 4.4. Zoledronate and Pravastatin Treatment (ZOPRA)

The combination (ZOPRA) of an anti-osteoporosis bisphosphonate (zoledronate) and an anti-cholesterolemic statin (pravastatin) has been shown to accelerate the RIANS [39]. The ZOPRA treatment was applied under conditions that are described elsewhere. Briefly, cells were incubated with 1 µM pravastatin (#P4498, Sigma-Aldrich France, Saint-Quentin-Fallavier, France) in phosphate-buffered saline (PBS) (#14040-091, Sigma-Aldrich) for 24 h at 37 °C. Thereafter, 1 µM zoledronate (#SML0223, Sigma-Aldrich) in PBS was added to the culture medium, and cells were incubated for 12 h at 37 °C [39]. 

### 4.5. Clonogenic Cell Survival

The intrinsic cellular radiosensitivity was quantified from clonogenic cell survival data obtained from standard delayed plating procedures that are described elsewhere [50]. Cells in the plateau phase of growth were irradiated at the indicated doses, incubated for 24 h at 37 °C, and then harvested, counted using a hemocytometer (Kisker Biotech GmbH & Co, Steinfurt, Germany), and diluted to a predefined number of cells (between 100 and 1000 cells) to be seeded in 10 cm Petri dishes. After 15 days at 37 °C in a CO_2_ incubator, cells were washed with PBS (Sigma-Aldrich) and stained for 1 min in crystal violet solution (75% ethanol 95%, 25% crystal violet #HT90132, Sigma-Aldrich). After a final wash with water, only the colonies with more than 50 cells were scored. The survival data were fitted to the linear quadratic (LQ) model that describes the cell survival S as a function of dose D as follows: $S=e^{-\left(\alpha D+\beta D^{2}\right)}$, where α and β are adjustable parameters to be determined. The intrinsic radiosensitivity was quantified by calculating the surviving fraction at 2 Gy (SF2) [44].

### 4.6. Immunofluorescence

The immunofluorescence protocol and nuclear protein foci scoring are described elsewhere [29,30]. Four antibodies against USH1 proteins were used at 1:100: the polyclonal anti-rabbit anti-cadherin like 23 (#ab131135), the monoclonal anti-rabbit anti-myosin VII A (#ab150386), the polyclonal anti-rabbit anti-USH1G (#ab150820), and the monoclonal anti-USH1C (#ab56812), all from Abcam (Cambridge, UK). Anti-*γH2AX^ser139^* antibody (#05-636; Merck Millipore, Burlington, VT, USA) was used at 1:800. The monoclonal anti-mouse anti-*MRE11* (#56211) from QED Bioscience (San Diego, CA, USA) and the monoclonal anti-mouse anti-*pATM^ser1981^* (#05-740) from Merck Millipore were used at 1:100. Incubations with anti-mouse fluorescein (FITC) and rhodamine (TRITC) secondary antibodies were performed at 1:100 at 37 °C for 20 min. Slides were mounted in 4′,6′diamidino-2-phenyl-indole (DAPI)-stained VECTASHIELD (CliniSciences, Nanterre, France) and examined with an Olympus BX51 fluorescence microscope. 

### 4.7. Proximity Ligation Assay

The proximity ligation assay (PLA) is a specific immunofluorescence technique that allows visualization of endogenous protein–protein interactions at the single molecule level [51]. Briefly, cells were plated on glass coverslips until reaching a minimum of 70% confluency. Cells were then fixed in 4% (*w/v*) paraformaldehyde for 15 min at room temperature, and were permeabilized in 0.5% Triton X-100 solution for 3 min at 4 °C. Cells were then blocked for 2 h at room temperature using 30 µL of blocking solution from the Duolink^TM^ In Situ Orange Starter Kit Mouse/Rabbit (#DUO92102, Sigma-Aldrich) per cover slip. Mixtures of two primary antibodies’ incubations were performed for 1 h at 37 °C. The following antibodies were all diluted in the Duolink antibody diluent 1X (#DUO82008, Sigma-Aldrich) at a ratio of 1:100: mouse monoclonal antibody (2C1 (1A1)) anti-ATM (#ab78), rabbit monoclonal (EPR7497), anti-Myosin VIIa/MYO7A (#ab150386), rabbit polyclonal anti-cadherin like 23 (#ab192498-Abcam), rabbit polyclonal anti-USH1G (#ab150820), and rabbit monoclonal anti-USH1C/Harmonin (#ab133763) from Abcam. PLA probes (Duolink PLA Probe anti-mouse MINUS #DUO82004-100RXN, Lot #SLCD468 and Duolink PLA Probe anti-rabbit PLUS #DUO82002-100RXN, Lot #SLLC564 from Sigma-Aldrich) were diluted using Duolink antibody diluent at a 1:5 ratio, and cells were incubated with the probes for 1 h at 37 °C in a humidified chamber. After 1 h of incubation, cells were washed with Wash Buffer A (#DUO82046-1EA; Sigma-Aldrich). Cells were then incubated with the ligation solution from the kit for 30 min at 37 °C in the humidified chamber, and then washed once more with Wash Buffer A. Cells were incubated with the amplification solution from the kit for 100 min at 37 °C in darkness and in the humidified chamber. Cells were then washed with Wash Buffer B (#DUO82048-1EA, Sigma-Aldrich), followed by a quick wash with 1/100 Wash Buffer B. Samples were mounted with Duolink In Situ Mounting Medium with DAPI (#DUO82040-Sigma-Aldrich), and subsequently analyzed under fluorescent microscopy. Fluorescent images were viewed under an Olympus BX51 microscope. Analysis and quantification of these samples were performed using ImageJ software. PLA dots were quantified on 16-bit images using the “Analyze Particles” command.

### 4.8. Cell Extracts and Immunoblots

Total extracts were obtained from cells with the following lysis buffer—50 mM Tris, pH 8, 150 mM NaCl, 2 mM EDTA, pH 8, 10% glycerol, 0.2% Nonidet NP40—and applied for 20 min at 4 °C. Cytoplasmic extracts were obtained from cells using the following buffer—10 mM HEPES pH 7.9, 1.5 mM MgCl2, 10 mM KCL, 2 mM ethylenediaminetetraacetic acid (EDTA) pH 8, 0.5 mM dithiothreitol (DTT), 0.2% Nonidet NP40, H_2_O)—applied for 15 min at 4 °C. Both buffers were supplemented with protease and phosphatase inhibitors (#78442, Thermo Fisher, Waltham, USA). Protein concentrations were measured with the Bio-Rad Bradford assay (Bio-Rad Laboratories, Hercules, CA, USA), and aliquots of extracts were stored at −20 °C. Proteins were subjected to SDS–polyacrylamide gel electrophoresis (SDS–PAGE) and blotted onto polyvinylidene fluoride (PVDF) membranes (Immobilon-P, Millipore). Membranes were blocked in Tris-buffered saline (TBS) solution containing 0.05% Tween 20 and 5% (*w/v*) non-fat dried skimmed milk powder, and incubated with primary antibodies for 3 h and with horseradish-peroxidase-conjugated secondary antibodies (Jackson ImmunoResearch, West Grove, PA, USA) for 1 h. Antibody binding was determined using the Clarity Max ECL substrate (#1705061, Bio-Rad Laboratories) and/or the SuperSignal West Femto Maximum Sensitivity Substrate (#34095, Thermo Scientific, Waltham, MA, USA). Western blot bands were analyzed using Image Lab software (Bio-Rad Laboratories, Hercules, CA, USA).

### 4.9. Statistical Analysis

The immunofluorescence data were fitted to the so-called Bodgi’s formula, which describes the kinetics for appearance/disappearance of nuclear foci formed by some protein relocalization after genotoxic stress [36]. Statistical analysis was performed by using KaleidaGraph v4 (Synergy Software, Reading, PA, USA) and GraphPad Prism (San Diego, CA, USA).

## 5. Conclusions

The USH1 syndrome appears to be a rare genetic syndrome associated with moderate but significant radiosensitivity, high yields of micronuclei, impaired DSB recognition but normal DSB repair, and delayed RIANS. This latter radiobiological feature is probably due to a facilitated interaction between ATM and the USH1 proteins—especially UHS1B/MYO7A—that are overexpressed in the cytoplasm in skin fibroblasts from USH1 patients (Figure 10). 

## Figures and Tables

**Table 1:** Major studies dealing with the radiobiological characterization of USH cells.

USH Cells	Techniques	Conclusions	Reference
Lymphoblastoid cells: GM03853, GM03892, RB4361, RB5062, RB5204, RB5207, RB5333, RB5360; Skin fibroblasts: GM03854, GM03889, GM03891.	Exclusion assay	Low radiosensitivity	[21]
Skin fibroblasts: GM03889, GM03854, GM03891.	Colony formation	Severe radiosensitivity	[20]
Skin fibroblasts: GM03889, GM03854, GM03891.	Colony formation γH2AX, pATM, and MRE11 foci	Moderate radiosensitivity	This study

## Data Availability

All of the data used in this work can be provided upon reasonable request.

## Supplementary Materials

The following supporting information can be downloaded at https://www.mdpi.com/article/10.3390/ijms23031570/s1.
