# Supplementary material for: Usher Syndrome Belongs to the Genetic Diseases Associated with Radiosensitivity: Influence of the ATM Protein Kinase
Source: Int J Mol Sci. 2022 Jan 29;23(3):1570. doi: 10.3390/ijms23031570 (PMC8836140; doi:10.3390/ijms23031570)
Supplement: Supplementary file 1 [file ijms-23-01570-s001.zip › ijms-1562296-supplementary.pdf]

Uher syndrome belongs to the genetic diseases associated with radiosensitivity: influence of the ATM protein kinase

Supplementary data

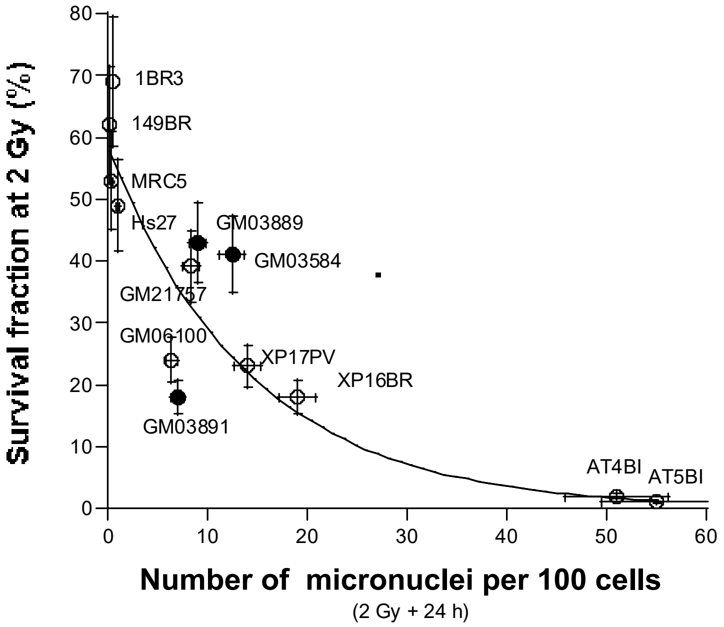

Figure S1 : Relationship between cellular radiosensitivity and residual micronuclei. Survival fraction at 2 Gy (SF2) data were plotted against the corresponding number of micronuclei per 100 cells assessed after 2 Gy X-rays following by 24 h post-irradiation time for the indicated cell lines. Data fit corresponds to the following formula : $y= 58.57*\exp(-0.07x)$ (r=0.95).

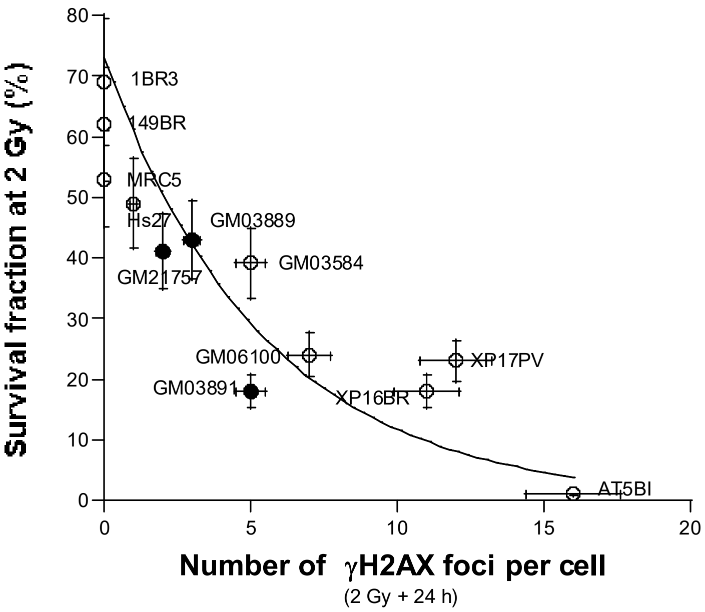

Figure S2 : Relationship between cellular radiosensitivity and residual γH2AX foci. Survival fraction at 2 Gy (SF2) data were plotted against the corresponding number of γH2AX foci assessed after 2 Gy X-rays following by 24 h post-irradiation time for the indicated cell lines. Data fit corresponds to the following fomula :y = 73.13*exp(-0.183x) (r=0.95).

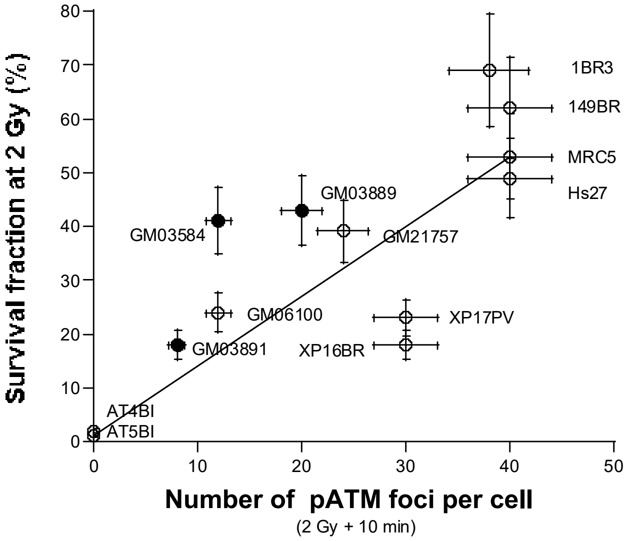

Figure S3 : Relationship between cellular radiosensitivity and early pATM foci. Survival fraction at 2 Gy (SF2) data were plotted against the corresponding number of pATM foci assessed after 2 Gy X-rays following by 10 mi post-irradiation time for the indicated cell lines. Data fit corresponds to the following fomula :y = 1.05 + 1.297x (r=0.86).
